# Body composition in preschool children with short stature: a case-control study

**DOI:** 10.1186/s12887-022-03159-8

**Published:** 2022-02-18

**Authors:** Yi-ting Ji, Li-li Li, Shi-zhong Cai, Xiao-yan Shi

**Affiliations:** Department of Child and Adolescent Healthcare, Children’s Hospital of Soochow University, Suzhou, 215021 Jiangsu China

**Keywords:** Short stature, Preschool children, Body composition, Case-control study

## Abstract

**Background:**

Short stature is defined as height below 2 standard deviations of the population with the same age, gender. This study is aimed to assess the characteristics of body composition in preschool children with short stature.

**Methods:**

Anthropometric measurements and body composition were assessed in 68 preschool children aged 3 to 6 years old with short stature and 68 normal controls matched on age and gender. Height, weight and body composition (total body water, protein, minerals, body fat mass, fat-free mass, soft lean mass, skeletal muscle mass, and bone mineral contents) in the two groups were measured and compared.

**Results:**

The total body water, protein, minerals, body fat mass, fat-free mass, soft lean mass, skeletal muscle mass, and bone mineral contents were lower in preschool children with short stature than controls (*P* < 0.05). Body mass index and fat mass index did not differ between groups. Fat-free mass index was significantly lower in short stature group than controls (t = 2.17, *P* = 0.03). Linear regression analysis showed that there was a positive correlation between height and fat-free mass index [β, 1.99 (0.59, 3.39), *P* = 0.01], a negative correlation between height and body fat percentage [β, − 0.20 (− 0.38, − 0.01), *P* = 0.04]. The proportions of fat-free mass in the upper limbs were significantly lower (Right,t = − 2.78,Left t = − 2.76, *P* < 0.05, respectively) in short stature, although body fat distribution was not.

**Conclusions:**

The fat-free mass such as protein and bone minerals is lower in preschool children with short stature, suggesting the monitoring of fat-free mass for early identification and intervention.

## Background

Growth can be affected by a number of factors, such as nutrition, culture, race and socio-economic conditions. Short stature is defined as height lower than minus two standard deviations(− 2 SDs) below the mean for chronologic age, gender, and racial or ethnic group [1]. The etiology includes constitutional growth delay, familial short stature, idiopathic short stature, abnormal pituitary development, chromosomal aberration, brain injury, or malnutrition [2]. Most cases of short stature children have no clear etiology or known genetic antecedent and are classified as idiopathic short stature, and cases present with normal levels of growth hormone. These cases may still exhibit abnormal receptor expression and endocrine axis function, as well as abnormal secretion of insulin-like growth factor 1 (IGF-1) and insulin-like growth factor binding protein 3 (IGFBP-3) [3]. The nutritional status of short stature in children may be thin, overweight, or obese,which can be measured by body mass index (BMI). BMI is a measured index of body on height and weight, widly-used to evaluate the body shape. However, BMI cannot distinguish between fat mass and fat-free mass, and does not reflect the distribution of body fat [4]. A comparison of the proportion of body fat mass and fat-free mass in children can better reflect body composition. Fat-free mass includes total water, protein, minerals, and other relatively healthy constituents and important tissues and organs such as bone and muscle, essential for growth and development [5]. In this study, we used the fat mass index (FMI) and fat-free mass index (FFMI) to represent the proportion of body fat mass and fat-free mass, which were included in body composition measurements. Therefore, in this study, we used BMI in tandem with FMI and FFMI to improve the comparison [6]. Bioelectrical impedance analysis (BIA) was used to calculate the proportion of body constituents such as water, fat, and protein by conductivity. In recent years, BIA has been widely applied to evaluate nutritional status, calculate the proportion and distribution of constituents of the human body, and study body composition measurements in children [7]. The objective of this study was to analyze the characteristics of body composition measurements in preschool children with short stature so as to assess their nutritional status and provide a reference for diagnosis, treatment, and dietary guideline.

## Methods

### Subjects and sample

**Fig. 1:**
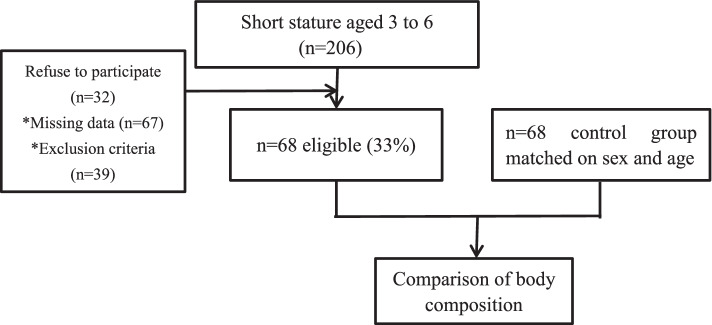
Screening of participants and recruitment flow. *Missing data: cannot complete the test of body composition or missing data around BMI. *Exclusion criteria: suspected abnormal development of skeletal system(*n* = 2); clinical status such as osteomalacia, severe chronic organic lesions, endocrine diseases, abnormal metabolic lesions, hypothyroidism, or chromosome abnormalities(*n* = 19); previously received drug treatments such as glucocorticoids and growth hormone(*n* = 10); BIA contraindication such as metal or electronic implants (atrial pulse generators, defibrillators, or pacemakers)(*n* = 8)

Preschool children aged 3 to 6 years with short stature were voluntary enrolled between September 2016 and March 2019 from the Department of Child and Adolescent Healthcare, Children’s Hospital of Soochow University in Suzhou, China. The study recruitment process is outlined in Fig. 1. Inclusion criteria: height below 2 SD of normal children of the same region, age and gender, and normal body weight and body length at birth. The bone age (BA) of all the participants was within the limits of 2 years of chronological age. Exclusion criteria: suspected abnormal development of skeletal system; clinical status such as osteomalacia, severe chronic organic lesions, endocrine diseases, abnormal metabolic lesions, hypothyroidism, or chromosome abnormalities; previously received drug treatments such as glucocorticoids and growth hormone; BIA contraindication such as metal or electronic implants (atrial pulse generators, defibrillators, or pacemakers). The control group with normal height was recruited randomly from the medical examination clinic during the same period. They were given thorough physical examination including detailed medical history and the previous growth curve was in the normal range. The subjects included 28 boys and 40 girls aged 3 to 6 years (mean: 4.76 ± 0.88 years). Sixty-eight children with normal height admitted to our department were selected as controls, matched on gender and age and with a mean age of 4.73 ± 0.89 years. Table 1 list subject characteristics. This study was approved by the Ethics Committee of the Children’s Hospital of Soochow University (Suzhou, Jiangsu, China). All parents or guardians of participants signed informed consent forms.

**Table 1:** Basic characteristics of short stature and control group

Group	Short stature group (*n* = 68)	Control group (*n* = 68)	*T/χ*^*2*^*/Z* value	*P* value
Age (years)	4.76 ± 0.88	4.73 ± 0.89	−0.17	0.86
Sex				
Male	28	28	–	–
Female	40	40		
Height (cm)	96.94 ± 5.18	107.78 ± 6.87	10.38	<0.05
Height-for-age z score median(P25,P75)	−2.37(− 2.53,-2.17)	−0.04(−0.60,0.66)	− 10.06	<0.05
Weight (kg)	14.08 ± 1.66	17.57 ± 2.64	9.23	<0.05
Weight-for-age z score median(P25,P75)	−1.73(−2.13,-1.34)	−0.02(− 0.85,0.36)	− 8.70	<0.05

t- test was used to compare normally distributed data (age, height, weight); and the Mann- Whitney U test was used to compare non-normally distributed data (Height-for-age z score, Weight-for-age z score)

### Anthropometric measurements and body composition

The height and weight of participants were measured by the same professional medical experimenter using the same digital portable scale (Betterren Co.Ltd., Shanghai, China). Participants were asked standing barefoot with eyes directed straight ahead. Height was measured to the nearest 0.1 cm and the accuracy of weight measurements was within 0.1 kg. Body composition was measured using the InBody J10 analyzer (InBody Co., Ltd., Seoul, Korea), which operated on the principle of bioelectrical impedance [8]. The indoor environment was maintained at 20–25 °C. The participants were measured after fasting, emptying their bladders, and resting quietly for 30 min. When measuring the body composition, the participants were asked to remove socks, carry no metal items, and stand in the correct position detailed by the manufacturer. Information concerning the participant (age, gender, and height) was entered by the experimenter. The body composition measurements included total body water, protein, minerals, body fat mass, fat-free mass, soft lean mass(made up of skeletal and smooth muscle), skeletal muscle mass, bone mineral content, and basal metabolic rate, which were automatically generated by InBody J10 analyzer.

BA was measured by taking X-ray of the left hand of the participants, including the hand bone, wrist, and radial ulnar stem 3–4 cm.

### BMI and the components

BMI (in kg/m^2^) was calculated with weight (kg) and height (m) measurements. The body fat mass represented the actual weight of fat in the body, while fat-free mass represented muscles, bones, tissues, and water. FMI and FFMI can be obtained from the analyzer data by dividing the body fat mass and fat-free mass by the height squared. Body fat percentage,basal metabolic rate and waist hip ratio were related to body composition. They were also calculated to represent fat distribution which automatically reported by the analyser.

### Quality control

Prior to the study, the same professional medical experimenter using the measuring tool was trained in the operation of the digital portable scale and InBody J10 analyzer. The instruments were standardized each day before the test. Anthropometric parameters, including height, weight and BMI of participants were converted to standard deviation scores (SD scores) as height-for-age z score, weight-for-age z score and BMI-for-age z score using the evaluation software (WHO Anthro 2005, [9]).

### Statistical analysis

SPSS V.21.0 statistical software (IBM Corp, Armonk, New York, USA) was used for data analysis. After testing the data distribution and variance homogeneity, the measurement data, such as height, weight,age, body composition measurements and other components were normally distributed variables and expressed as the mean ± SD. Comparisons between two groups were made using independent-sample t- tests. Height-for-age z score, weight-for-age z score and BMI-for-age z score were non-normally distributed variables and expressed as median (25th percentile, 75th percentile) and the non-parametric Wilcoxon Mann-Whitney test was used between two groups. The classification data, such as sex, were expressed as numbers and compared by χ^2^ test. Statistical significance was set at a *p* < 0.05.

## Results

### Basic characteristics of short stature and control group

No significant difference was observed in age or sex between the short stature group and the control group (*P* > 0.05). The height (height-for-age z score) and weight (weight-for-age z score) of participants in short stature group were significantly lower than those in the control group (*P* < 0.05) (Table 1).

### Comparison of body composition measurements between two groups

Total water, protein, minerals, fat mass, fat-free mass, muscle mass, skeletal muscle, and bone mineral contents were significantly lower in the short stature group than in the controls (*P* < 0.05) (Table 2).

**Table 2:** Comparison of Body composition measurements between two groups (kg)

Group	Short stature group (*n* = 68)	Control group (*n* = 68)	*T* value	*P* value
Total water	8.38 ± 1.48	10.64 ± 1.65	8.43	<0.05
Protein	2.23 ± 0.38	2.81 ± 0.45	8.21	<0.05
Minerals	0.74 ± 0.46	0.97 ± 0.40	3.02	<0.05
Fat mass	2.74 ± 1.11	3.13 ± 1.18	1.98	<0.05
Fat-free mass	11.34 ± 1.75	14.44 ± 2.25	8.97	<0.05
Soft lean mass	10.74 ± 1.89	13.64 ± 2.11	8.42	<0.05
Skeletal muscle mass	4.71 ± 1.20	6.54 ± 1.34	8.37	<0.05
Bone mineral content	0.60 ± 0.46	0.79 ± 0.38	2.66	<0.05

t- test was used to compare normally distributed data

### Comparison of BMI and other components between two groups

BMI (BMI-for-age z score) and FMI did not differ between groups, but FFMI was significantly lower in short stature group than in the controls (*t* = 2.17, *P* = 0.03). Body fat percentage and the waist-to-hip ratio did not differ between groups (*t* = 1.56, *P* = 0.12) (Table 3).

**Table 3:** Comparison of BMI and other components between two groups

Group	Short stature group (*n* = 68)	Control group (*n* = 68)	*T* value	*P* value
BMI (kg/m^2^)	14.96 ± 0.98	15.04 ± 1.00	0.51	0.61
BMI-for-age z score	−0.23(− 0.79,0.43)	−0.02(− 0.85,0.36)	− 0.55	0.58
FMI (kg/m^2^)	2.95 ± 1.32	2.69 ± 1.01	−1.30	0.19
FFMI (kg/m^2^)	12.00 ± 1.06	12.36 ± 0.81	2.17	0.03
Body fat percentage (%)	19.53 ± 7.89	17.65 ± 6.07	−1.56	0.12
Basal metabolic rate (KJ/h)	614.74 ± 37.77	681.85 ± 48.48	9.00	<0.05
Waist hip ratio (%)	0.69 ± 0.03	0.68 ± 0.03	−0.44	0.66

*Abbreviation*:*BMI* Body mass index, *FFMI* Fat mass index, *FMI* Fat-free mass index. t- test was used to compare normally distributed data

### Effects of BMI and other components on height

To explore the effect of BMI and other components on height for further exploration. Designating the height of all the participants as dependent variables and BMI(adjusted age and sex), FFMI (adjusted age, sex and FMI), FMI (adjusted age, sex and FFMI) and Body fat percentage (adjusted age, sex and BMI) as independent variables, linear regression analysis showed that there was a positive correlation between height and FFMI [β Estimate, 1.99 (0.59, 3.39), *P* = 0.01], a negative correlation between height and body fat percentage [β Estimate, − 0.20 (− 0.38, − 0.01), *P* = 0.04] (Table 4).

**Table 4:** Effects of BMI and other components on height

Variables	*β* Estimate (95%CI)	*T* value	*P* value
BMI^a^	1.03 (− 0.07, 2.12)	1.85	0.07
FFMI^b^	1.99 (0.59, 3.39)	2.80	0.01
FMI^c^	0.67 (−0.46, 1.81)	1.18	0.24
Body fat percentage^d^	−0.20 (− 0.38, − 0.01)	−2.13	0.04

*Abbreviation*: *BMI* Body mass index, *FFMI* Fat mass index, *FMI* Fat-free mass index. ^a^, adjusted age and sex; ^b^, adjusted age, sex and FMI; ^c^, adjusted age, sex and FFMI; ^d^, adjusted age, sex and BMI

### Distribution of body fat mass and fat-free mass in short stature group

No significant difference was observed in the distribution of body fat in the upper and lower limbs or the buttocks between short stature group and in the controls (*P* > 0.05). The distribution of fat-free mass in both upper limbs of short stature group was significantly lower than in the controls (Right t = − 2.78,Left t = − 2.76, *P* < 0.05) but the distribution in the lower limbs and buttocks was not (*P* > 0.05).

## Discussion

Human body is mainly composed of water, protein, minerals, and fat. A reasonable distribution of the constituents within children can ensure their health and nutritional balance [10].BIA is a good measure of body composition because it is radiation-free, noninvasive testing and easy to perform. It has a high consistency with standard dual-energy X-ray absorptiometry [11, 12]. However, there have been few reports to date on body composition in short stature, especially preschool children aged 3 to 6.

60–80% of children with short stature do not exhibit systemic, endocrine, nutritional diseases or chromosomal abnormalities and have no deficiency in growth hormone [13]. The etiology remains unknown, and may involve the interaction between height-related genes and the environment. Some studies have suggested that.

a heterozygous mutation or deletion of short stature homeobox (SHOX) gene is a rare cause of short stature [14, 15]. In 2003, the US Food and Drug Administration approved the use of recombinant human growth hormone (rhGH) in idiopathic short stature children [1, 16], and clinical trials have been conducted in China [17]. In addition, nutritional status and body composition in children with short stature should be a focus of early detection and diagnosis of disease. The nutritional health and physical condition of children can be assessed from body composition at various growth and development stages; certain diseases may promote abnormal body composition and affect growth [18, 19].

Height is a long-term index of nutritional and growth status, perhaps owing to early malnutrition, rearing behavior, or family and social environment. In this study, we found differences of fat-free mass such as protein and minerals between short stature and normal controls with similar physical conditions, suggesting abnormal nutrition in short stature. Therefore, we recommend an increasing intake of protein and minerals to preschool children with short stature. Studies have shown that short stature children were prone to complications from nutritional metabolic disorders such as malnutrition and obesity, which were associated with IGF-1 and IGFBP-3 [3, 20]. In this study, no significant difference was found in BMI between short stature and controls, indicating that they have similar overall figure. Unlike our outcomes, some previous studies suggested that short stature children have a higher incidence of obesity; especially in short stature with growth hormone deficiency, their glucose and lipid metabolism can be impaired, resulting in insulin resistance and cardiovascular disease [21].

A population study in Mexican found that different criteria of BMI and body fat percentage in high or short stature distinguished obesity, which meant that different body proportions may have different body fat distribution [22]. Previous studies have shown that the incidence of overweight and obesity was higher in taller children [20, 23]; these studies compared BMI and body fat percentage, which was related to infant nutritional status, childhood growth hormone and adolescent gonadal hormone levels, and genetic factors. In this study, although no differences were found in BMI, FMI, or body fat percentage between short stature and the controls. FFMI, which represented the proportion of healthy body constituents, was lower in short stature. The linear regression analysis was consistent with the previous outcome. FFMI and body fat percentage were independent influencing factors that affected the height after adjusting age, sex and FMI or BMI respectively. Segmental distribution showed that the content of protein and minerals in the upper limbs of short stature was lower, indicating reduced bone and muscle growth. Therefore, preschool children with short stature should be encouraged to engage in physical exercise so as to strengthen their upper limbs and increase their fat-free mass. The basal metabolic rate is the lowest energy needed for the normal daily operation of the body under rest, and fat-free mass influences 80% of this rate [4]. We found that the basal metabolic rate was lower in short stature than in normal children, mainly because of the difference in their fat-free mass. The children in our cohort had no abnormalities in endocrine factors such as growth hormone. In preschool children, early nutrition, exercise, and hormone levels may affect height, and early intervention and nutrition guidance can improve their final adult height (FAH).

This study had some limitations. We limited age range from 3 to 6 years to exclude the influence of pubertal development on body composition, and other age groups should be investigated as well. In addition, we did not measure the plicometric assessments and laboratory parameters such as the levels of growth hormone, IGF-1, IGFBP-3, insulin, and other blood lipids, which should be measured in the follow-up studies. There were some influencing factors that cause different outcome, such as fasting or eating status and measurement at the different time points [24]. Finally, there were no overweight or obese children in our cohort, which may be related to the small sample size and to population selection bias.

## Conclusions

Fat-free mass, such as protein and bone minerals, are lower in preschool children with short stature, indicating higher risks of developing nutritional and metabolic abnormalities. Clinicians may find it useful to assess the distribution of body composition so as to give guidelines to caregivers in the management of nutrition and exercise in preschool children with short stature.

## Data Availability

The datasets used and/or analysed during the current study available from the corresponding author on reasonable request.

## Abbreviations

BA: Bone age
BIA: Bioelectrical impedance analysis
BMI: Body mass index
FAH: Final adult height
FFMI: Fat-free mass index
FMI: Fat mass index
IGF-1: Insulin-like growth factor 1
IGFBP-3: Insulin-like growth factor binding protein 3
rhGH: Recombinant human growth hormone
SHOX: Short stature homeobox
